# Enrichment of OpenStreetMap Data Completeness with Sidewalk Geometries Using Data Mining Techniques

**DOI:** 10.3390/s18020509

**Published:** 2018-02-08

**Authors:** Amin Mobasheri, Haosheng Huang, Lívia Castro Degrossi, Alexander Zipf

**Affiliations:** 1 GIScience Research Group, Institute of Geography, Heidelberg University, 69120 Heidelberg, Germany; zipf@uni-heidelberg.de; 2 GIScience Center of the Department of Geography, University of Zurich (UZH), 8057 Zurich, Switzerland; haosheng.huang@geo.uzh.ch; 3 Department of Computer Systems, University of São Paulo, São Carlos 13566-590, Brazil; liviadegrossi@gmail.com

**Keywords:** sidewalk, routing, open data, OpenStreetMap, data quality, completeness

## Abstract

Tailored routing and navigation services utilized by wheelchair users require certain information about sidewalk geometries and their attributes to execute efficiently. Except some minor regions/cities, such detailed information is not present in current versions of crowdsourced mapping databases including OpenStreetMap. CAP4Access European project aimed to use (and enrich) OpenStreetMap for making it fit to the purpose of wheelchair routing. In this respect, this study presents a modified methodology based on data mining techniques for constructing sidewalk geometries using multiple GPS traces collected by wheelchair users during an urban travel experiment. The derived sidewalk geometries can be used to enrich OpenStreetMap to support wheelchair routing. The proposed method was applied to a case study in Heidelberg, Germany. The constructed sidewalk geometries were compared to an official reference dataset (“ground truth dataset”). The case study shows that the constructed sidewalk network overlays with 96% of the official reference dataset. Furthermore, in terms of positional accuracy, a low Root Mean Square Error (RMSE) value (0.93 m) is achieved. The article presents our discussion on the results as well as the conclusion and future research directions.

## 1. Introduction

OpenStreetMap (OSM) is an example of Volunteered Geographic Information (VGI) project launched in 2004 [1]. VGI projects are those that aim to capture and provide information about the world by volunteers and the collected spatial information is freely made available [2]. Based on the nature of VGI projects, geographic information is collected and submitted by volunteers who are not necessarily familiar with (geo-)data collection procedures. This leads to strong concerns regarding the quality of the gathered information [3]. Several research studies have been conducted to understand and evaluate the quality of OSM data based on different data quality elements and for different application purposes [4]. Such studies include assessing the positional accuracy of road network [5,6] and building features [7], as well as the completeness of OSM regarding road network [5,8,9,10], sidewalk [11,12], and land use information [13]. In addition, several transportation (routing and navigation) studies deal with employing and analyzing VGI (and in particular OSM) as their primary data source [14,15,16,17,18,19,20]. This is because crowdsourced datasets tend to be up-to-date, especially in densely populated urban areas.

Our general research focuses on employing OSM data for wheelchair routing service. Quality of routing and navigation services relies on the quality of input datasets. Specialized routing systems such as those designed to guide user with restricted mobility need specific information regarding the road networks. The most important information for such services is sidewalk geometries and their detailed information such as sidewalk width, incline, surface texture, etc. Existing commercial or governmental geo-data sources do not usually contain this kind of information, and therefore are not able to support routing services for people with restricted mobility (e.g., wheelchair users).

Within the CAP4Access project (http://www.geog.uni-heidelberg.de/gis/cap4access_en.html), we aim to use OpenStreetMap data to provide routing and navigation services for people with limited mobility. In prior studies and analyses, it is understood that OSM data are largely incomplete when it comes to data about sidewalks [12]. Therefore, to enrich OSM data and make it a proper dataset for pedestrian/wheelchair routing services, it is crucial to define methods for the construction of sidewalk geometry. Different options could be used for this purpose such as computer vision techniques [21], Lidar data collection, mining for sidewalk construction [22], etc. However, these methods require access to many images or data, which are expensive. In this article, we propose an efficient method to use freely available data to overcome this issue. Specifically, we employ GPS traces by wheelchair volunteers, as well as road network dataset and building footprints both available in OSM database to derive sidewalk geometries. This can be used to enrich OSM data with sidewalk features and can be further used for routing and navigation of pedestrians and people who strongly require information on sidewalk availability for their travels (e.g., wheelchair users).

In the next section, we present the prior research studies that have been conducted, related to our study. In Section 3, our proposed methodology is introduced. Section 4 includes the results on data mining and analyses as well as evaluating the quality of the results. Finally, Section 5 concludes the paper and provides a future outlook for this study.

## 2. Related Studies

Sidewalk network datasets are in the heart of a database for wheelchair/pedestrian routing and navigation service. Recent advancement in GPS-enabled mobile technology has increased interest in pedestrian and wheelchair navigation services. Current routing and navigation systems such as the one developed and maintained by Google, support navigation for pedestrians but the datasets used for generating the routes are based on road networks and do not include sidewalk information [23]. This makes such services inefficient for sidewalk navigation because road networks usually model road centerlines and do not adequately address the sidewalk navigation environment [24,25]. For wheelchair routing services to provide effective routing and navigation assistance to pedestrians and people with reduced mobility, sidewalk networks are required to be modeled [12,24,26,27]. Although there is a strong demand for sidewalk network databases, these are not publicly available and until now only few attempts have been made to map sidewalk information in OpenStreetMap. More importantly, less attention has been paid to define and use a systematic approach for wide collection and construction of sidewalk datasets.

Several studies have focused on modeling sidewalk networks. Laakso et al. present a formal information model for pedestrian networks [28,29]. The authors emphasize on guidelines for data content and classification of map information that is more useful for people with restricted mobility and makes the environment more accessible [28]. Their model covers other geo-information that helps modeling the accessibility characteristics of the sidewalk network. The model aims to assist data providers in collecting and storing relevant data using efficient methods [29]. In another study [30], Beale and colleagues identify and quantify the differences between barriers to effective navigation such as slope or dropped curbs for able-bodied pedestrians and wheelchair users. This trend is followed and improved by Karimi et al. [31], where the authors have presented a personalized accessibility map (PAM) via geo-crowdsourcing. A prototype of PAM is developed and analyzed in detail which greatly helps in understanding the characteristics of such systems as well as the works that needs to be done in future studies.

Collaborative mapping is the approach of using sensors installed on mobile devices as well as web 2.0 technology that turns ordinary people into mapmakers [32]. Users of collaborative mapping platforms are provided with necessary tools and information on how to contribute to the project. Collaborative mapping allows us to develop simple and cost-effective approaches for collecting relevant information for modeling the required datasets (e.g., sidewalk network). Compared to traditional spatial data collection procedures that use advanced equipment and are disseminated by mapping agencies (national, commercial, etc.), crowdsourcing is a cheap approach which is subject to open licensing terms.

Nowadays, there are several studies on analyzing raw GPS data and extracting useful information from it. For instance, some studies investigate human behavior and mobility [33,34], understanding transportation modes [35,36] as well as journey planning [37] thorough analyzing GPS traces. In other studies [38,39], crowdsourced GPS data from cycling activities are used to understand hotspots of their destinations and their cycling behavior. Other relevant efforts deal with updating existing maps with new information retrieved from analyzing GPS data [40,41,42]. Furthermore, only few studies have so far dealt with employing GPS data for extracting sidewalk information relevant for wheelchair/pedestrian routing [43,44,45,46,47]. While such efforts exist, there is still great need of further research studies to suggest other methods or extend existing algorithms for deriving results that are more accurate.

In this article, similar to Kasemsuppakorn et al. [45], we explore the feasibility of using multiple GPS traces collected through collaborative activities to derive sidewalk geometries and further construct the sidewalk network. The difference between our work and the work proposed by [45] is that our algorithm considers extra data sources available in OSM database, i.e., road network and building footprints, to increase the positional accuracy of the generated sidewalk geometries. A main limitation using GPS data is that, due to the multipath problem with GPS, as wheelchair users navigate close to buildings, determining the geometry of a sidewalk by solely using GPS traces is unreliable. Moreover, GPS data recorded in different times along the same path may lead to different accuracies. To overcome this issue, we employ multiple GPS traces on the same path enhanced with road and building data directly available in OSM to minimize the errors caused by GPS. Therefore, it is expected that compared to [45], the proposed method in this study will lead to better positional accuracy of the constructed sidewalk geometries, especially in dense urban areas where GPS accuracy is rather poor.

It is worth noting that employing crowdsourced datasets for accessibility is a hot topic and various on-going efforts are happening in the current decade [48,49,50,51,52,53,54,55]. Our study is also in line with this trend and tries to address an existing gap in the field by answering to the question of how crowdsourced geographic information such as OpenStreetMap could be enriched to better serve a wheelchair routing engine. 

## 3. Methodology

**Figure 1:**
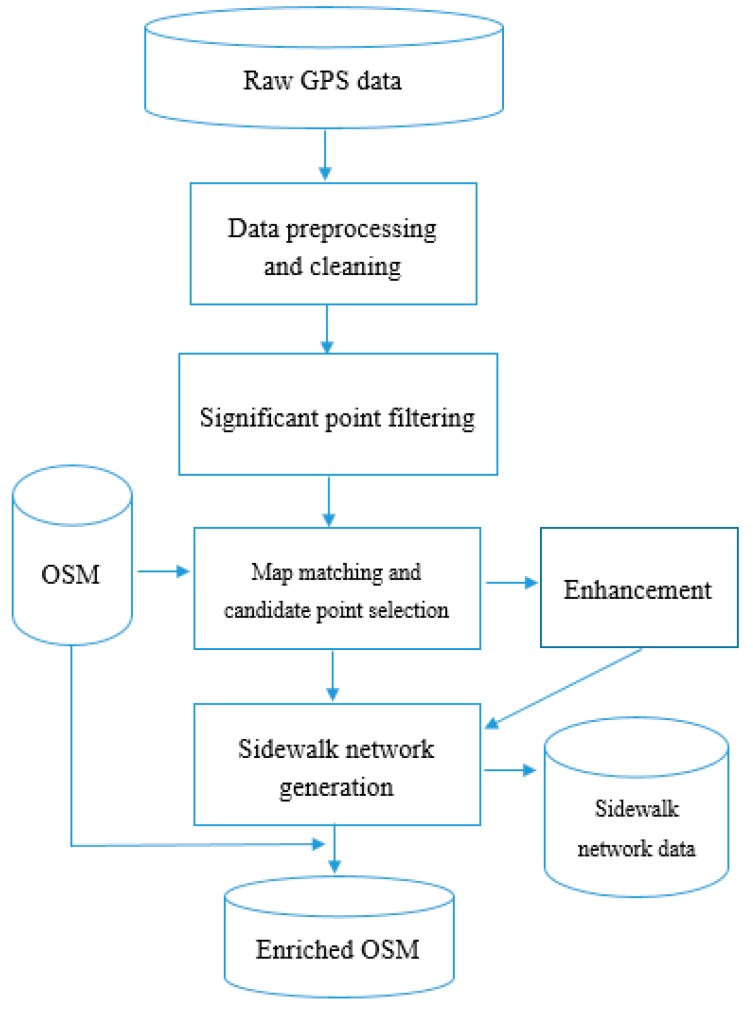
Workflow of the methodology.

In this section, we discuss an algorithm to construct a sidewalk network using multiple GPS traces contributed by volunteered wheelchair users. We mainly focus on the geometries of the sidewalks. A GPS trace refers to a trajectory of a wheelchair user traveling along pedestrian paths as recorded by a GPS receiver. We assume that the GPS traces represent the sidewalk segments traveled. The algorithm processes the GPS traces and has the following five steps: (1) pre-processing and cleaning; (2) significant point filtering; (3) map matching and candidate point selection; (4) enhancement; and (5) sidewalk network construction. The constructed sidewalk network can be then integrated to enrich OpenStreetMap data. Figure 1 highlights the input, the five steps, and the output of the algorithm. The first two steps are concerned with the processing of individual GPS traces based on the point-to-point property. Steps (3) and (4) use road centerlines as well as building footprints to increase the positional accuracy of the constructed sidewalk geometry. The fifth step “sidewalk network construction” is concerned with incorporating new input traces to the already constructed paths stored in the database (which is empty in the first place).

### 3.1. Data Preprocessing and Cleaning

The pre-processing step cleans raw GPS traces that usually contain errors due to uncertainty in the location fixes, the GPS Time-To-First-Fix (TTFF) problem, and the obscured GPS satellite signals where the satellites are obscured by buildings or dense tree canopy. In this step, GPS information, including latitude, longitude, time, speed, Horizontal Dilution of Precision (HDOP), and number of used satellites, is extracted. The filter considers GPS observations with less than four satellites and HDOP greater than a threshold (value 5, where positional measurements could be used to make reliable in-route navigation suggestions to the user) as outliers and eliminates them. To address the cold-start/TTFF problem, this step also eliminates all the GPS points that are recorded within the first 15 min when the GPS receivers are powered up. The results of this step are cleaned GPS traces without outliers. 

### 3.2. Significant Point Filtering

In this step, points of the cleaned GPS traces (from Section 3.1) are processed to identify the points that contain the most important characteristics with regard to the geometry of the traces (i.e., significant points). For example, a GPS trace of a straight line would only need the start and ending points to maintain the geometry, and therefore they are significant points. However, this is the simplest example. Identifying the significant points of curved paths from GPS traces is more challenging. In the following, we propose a method to deal with this issue. Specifically, each filtered GPS trace from Section 3.1 is further analyzed and processed in the following order:

(1) the bearing change (Δα) is calculated. The bearing of successive points in a filtered GPS trace is required to calculate the bearing change. The bearing information provided directly by the GPS receivers are not employed in this task due to lack of accuracy when traveling at speeds of less than 3.0 m/s [56]. Instead, we adopt the great circle navigation formula [57] for calculating the absolute value obtained from subtracting successive bearings (Δα).
(2) Each GPS point now contains the bearing between two successive points and the bearing change (Δα). Since the bearing change could have a value between 0 to 360 degrees, setting a threshold for selecting the candidate significant point based on the bearing change would be difficult. For instance, there might be a situation where the differences between the values of the two numbers is very high but the change in direction is not. Therefore, an algorithm for recognizing shapes of objects is necessary to be used. In our approach, we employ the chain coding technique, since it has been proven to work well for detecting sidewalk geometries [45]. For detailed information on this technique, please refer to [58].

**Figure 2:**
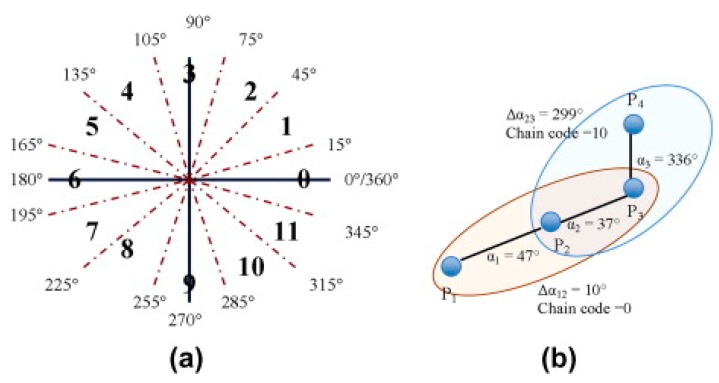
The 12-direction chain code (**a**); and an example (**b**), adopted from [38].

A 12-direction chain code is chosen to represent bearing change in 12 direction intervals. This is based on a counterclockwise direction from the positive *x*-axis. This enables us to model angle of turns. Figure 2 shows the 12-direction chain code (Figure 2a) and an example (Figure 2b).

After this step, the following GPS points are extracted for each GPS trace: start, end, and significant points. These points will be further refined and clustered in the next steps. 

### 3.3. Map Matching and Candidate Point Selection

Since the number of significant points might still be large, and to improve the efficiency of the algorithm (removing redundant points), further filtering is required. Therefore, we apply a clustering approach to group the significant points into different clusters using OpenStreetMap road network data and building footprints, and further select representative points of each cluster. Specifically, we use the OSM road and building data for the same area of GPS traces and follow two steps:

1. Calculate the distance of each GPS significant point with the nearest road line segment, and/or nearest building segment (this means that firstly we select a road or building object and then calculate the distance of it to all significant points and repeat it for all road or building objects in the area that traces overlap with). Then, we group those significant points that seem to have the similar distance to a road or building (this shows that the group of significant points belong to a path near either the road or the building). Hence, clusters for all the significant points are created. All the significant points need to be in at least one cluster in the end. This task is repeated until the clustering of all the points are processed.
2. For each cluster, the algorithm checks the value of the distance of points to nearest road/building, and selects three points from each cluster. Two of the points belong to the head and tail of the cluster (geographically located start and end points). The third point is the representative point of the cluster; hence, it is the point that has the closest distance to the correspondence road or building object. This step is repeated for all the clusters.

After the step, the significant points of each GPS trace from Section 3.3 are further filtered to a smaller number of representative points for further refinement, which can be used to construct sidewalk geometries. 

### 3.4. Enhancement

Furthermore, to improve the positional accuracy of GPS points (and the final sidewalk geometries), we perform an alignment process of successive GPS points that their position has been slightly changed comparing to its nearest road segment. This alignment procedure involves simple assignment of individual significant GPS point with its nearest road segment, and a shifting of location of GPS point so it falls within the acceptable range (if the data about the building footprint and road network are available in OSM). Section 4 will illustrate the results of such enhancement step in the case study.

### 3.5. Sidewalk Network Construction, and OSM Data Enrichment

Initially, the database contains road network and building geometries and the sidewalk network is empty. It is expected that over time the sidewalk data in the database are to be extended by newly collected and analyzed GPS traces. The input of the sidewalk construction is the significant points of a new GPS trace, obtained from the previous step, and the final output is the generated sidewalk network. The sidewalk construction step begins by loading a new set of GPS significant points (derived from Section 3.3), and defining its map boundary. Every trace is processed for three cases: (a) if the sidewalk geometry for that path does not exists, a new sidewalk segment is generated and loaded into database (i.e., data enrichment); (b) merge the new sidewalk segment within the existing sidewalk network; and (c) if the sidewalk geometry for that path exists, they should be compared and the geometry should be merged/updated (only if a change is seen). Please note that the enrichment step is done on local database and not on the original OSM database, since OSM does not allow bulk editing. Therefore, Figure 1 shows that sidewalk network dataset is created from the sidewalk network generation step.

## 4. Experiment and Results

### 4.1. Study Area

**Figure 3:**
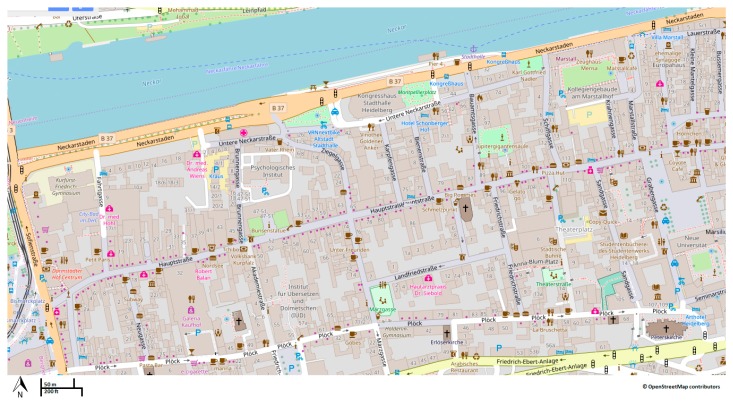
Part of Heidelberg old town where the experiment took place (map: © OpenStreetMap contributors).

**Figure 4:**
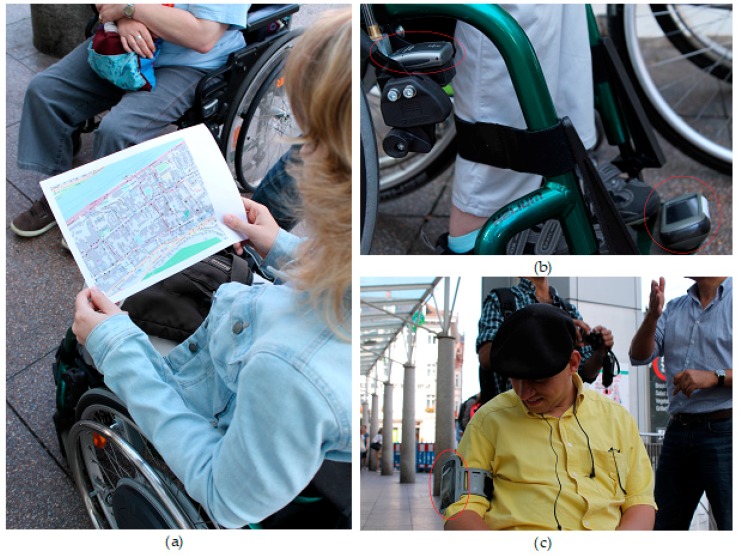
The navigation experiment: (**a**) a wheelchair user checking the navigation plan; (**b**) two GPS devices installed on wheelchair; and (**c**) GPS device worn by a user.

We have selected the main part of the old town in Heidelberg as a case study, since it is the main area for shopping and tourist attraction and through an initial checking of OSM data, it seems that the sidewalk data of OSM in this area is incomplete [12]. Figure 3 shows the area of interest. A navigation experiment with real wheelchair users was carried out and a total number of seven GPS receivers were installed on five wheelchairs. The GPS sensors were installed on different places of the wheelchair (Figure 4b) as well as the on the body of the users (Figure 4c). The wheelchair users were asked to navigate through the city according to the given map and agenda (Figure 4a). The path of data collection was chosen based on two aims: (a) the path should contain areas that sidewalks exists; and (b) the sidewalks should have different physical characteristics such as different surface texture, slope, etc. While the research done in this article only relies on the first reason for choosing the given path, we carried out the experiment for other research that involves understanding different characteristics of the sidewalks, and for that reason the second aim was considered relevant. 

### 4.2. Sidewalk Geometry Construction

In this section, we apply the methodology introduced in Section 3 for constructing a sidewalk network using multiple GPS traces collected by individuals on wheelchairs. A GPS trace refers to a trajectory of a wheelchair user traveling along pedestrian paths as recorded by a GPS receiver. Our assumptions are that the GPS traces represent the sidewalk segments traveled; each wheelchair user may provide more than one trace at different times; and, over time, each sidewalk segment is covered by multiple GPS traces. Five wheelchair users were involved in the experiment, with different number of GPS devices installed on each of them. Seven individual GPS traces were collected and used to perform our study.

#### 4.2.1. Preprocessing Step

**Figure 5:**
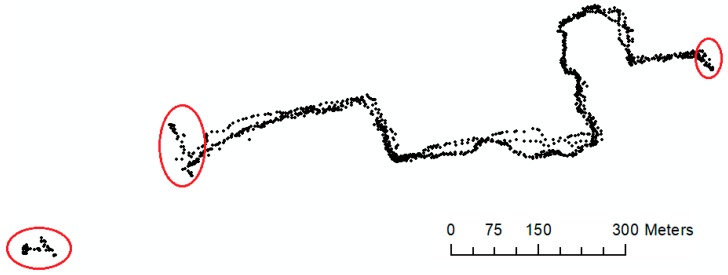
Raw GPS data before preprocessing—1559 points, and 287 GPS points counted as noise (in red circles).

In the first step, as explained in the Methodology, we extracted several useful information from each GPS trace record. This information included latitude, longitude, time, speed, etc. Such information is necessary to extract to be used later in the sidewalk construction algorithm. Furthermore, we excluded the first collected GPS points that have stored positions by GPS receivers as well as repetitive points of the last location by using the pre-processing method describe in Section 3.1. Figure 5 shows a snapshot of the raw GPS data prior to preprocessing as well as the result of pre-processed GPS data.

#### 4.2.2. Data Clustering and Candidate Point Selection

**Figure 6:**
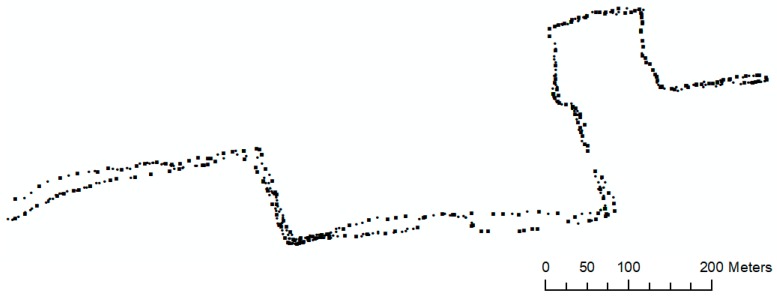
GPS points that shape the geometry of sidewalks (dots are clustered points (512), and squares are candidate points (267)).

As discussed earlier in Section 3.2, in this step, we identify the GPS points that contain the most important information about the geometry of the underlying individual traces. The analyses that have been performed in this step include calculating the bearing change as well as using the chain coding technique for identification of the most relevant points that shape the geometry of sidewalks. Figure 6 depicts the result of this step.

#### 4.2.3. Map Matching and Significant Point Selection

**Figure 7:**
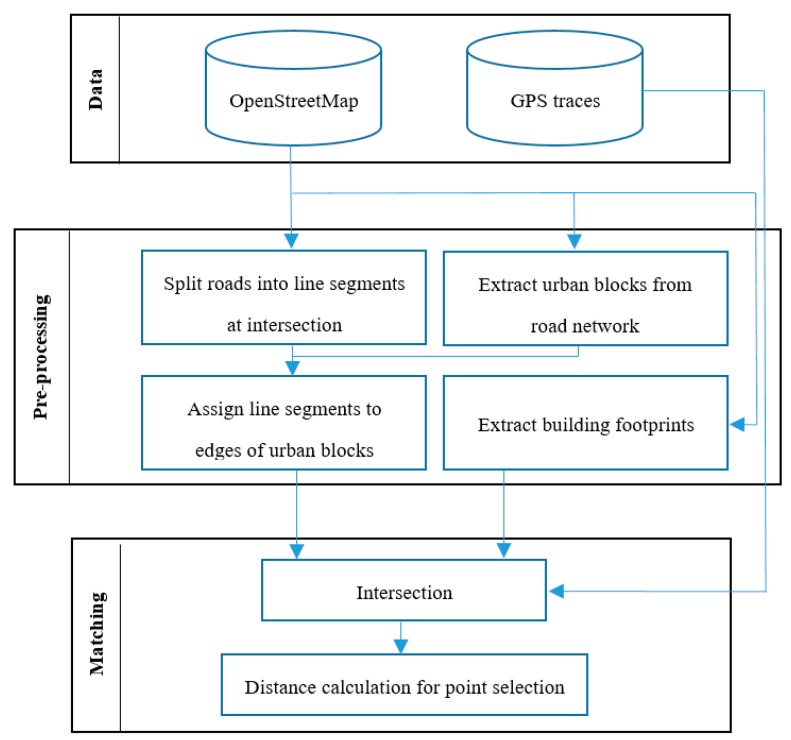
Workflow for matching and comparison of OpenStreetMap and GPS dataset.

As introduced in Section 3.3, in this step, we match the derived GPS candidate points with the road network and building data of OpenStreetMap using the map matching technique depicted in Figure 7. For matching OSM road features and buildings with the GPS data, we have adopted and extended the map matching approach presented by Fan et al. [59]. Figure 7 shows the flowchart of the tasks that were carried out for the map matching task.

**Figure 8:**
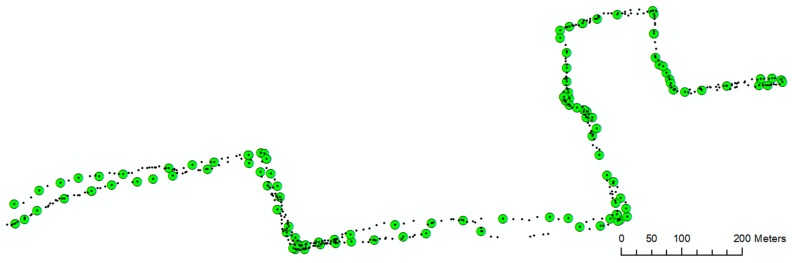
The final selected significant points (123 points).

As depicted in Figure 7, in the first stage the road segments in OSM data are split into various lines using their intersections. These line segments and building footprints are used for calculating the Euclidean distance of each GPS point with them. The process is repeated for all GPS points and all surrounding road lines segments and building footprints. In the final stage, those GPS points which fall in the acceptable range between the line segments and building footprints would be selected as a significant point, and those GPS points which fall outside the acceptable range are excluded. A challenge for this task was to identify a proper acceptance range for sidewalks. This range should logically be adapted considering the urban and transportation structure of a city (or part of a city). For this purpose, since our experiment was located in a dense urban environment, and based on the guidelines of city and urban structure, the proper acceptable range for construction of sidewalks in that area was configured as 1-m distance from building footprint and 3 m distance from road centerline. Based on this configuration, the selected significant points for constructing the sidewalk in our experiment was derived (Figure 8). 

#### 4.2.4. Enhancement

**Figure 9:**
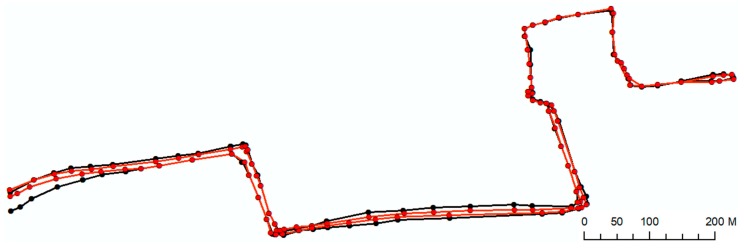
Comparison of the original (black) and the enhanced (red) constructed sidewalk network.

As mentioned in Section 3.4, enhancement of results is carried out by aligning GPS points through checking their distances with its nearest road segment. This procedure involves a shifting of location of GPS point so it falls within an acceptable range between road and buildings where the sidewalks actually exist in reality. Please note that, in the case of lack of road or building footprint information for a certain area, this stage is skipped. Figure 9 shows the enhanced sidewalk network geometry in red.

#### 4.2.5. Sidewalk Network Construction

As the final step of this process, the significant points extracted from each trace were used as input to the sidewalk network construction step. We applied the method introduced in Section 3.4 to connect the significant GPS points to derive the sidewalk geometry. Figure 9 shows the sidewalk generated from connecting the points presented in Figure 8 (significant points) in black. We further evaluate our approach in the next section.

### 4.3. Evaluation

The evaluation of the results was carried out in two ways. First, we performed a visual analysis of positional accuracy of the constructed sidewalk network. Later, this is carried out by comparing it with a reference dataset of sidewalks from the municipality of Heidelberg. To validate our approach in enhancing the results of sidewalk construction (Section 3.4.), we perform our evaluation in two different stages, prior to and after the enhancement stage.

#### 4.3.1. Visual Inspection with Google Maps

**Figure 10:**
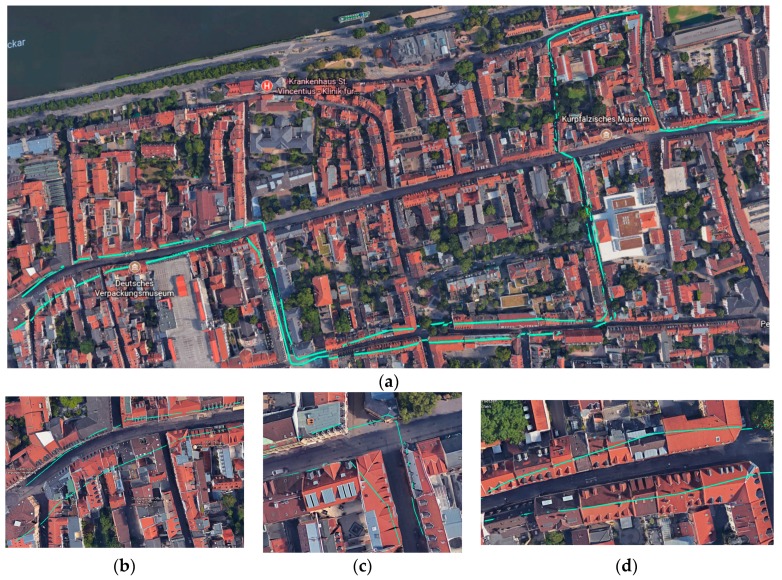
Results of visual inspection of sidewalk network with Google Maps (before applying the enhancement stage): (**a**) the overlaid sidewalk network for the whole experiment area; and (**b**–**d**) examples of positional inaccuracies.

As a basic approach to validate and evaluate our approach, we overlaid the generated sidewalk network on Google Maps. Figure 10a shows the overlaid sidewalk network for the whole experiment area. Figure 10b–d shows three examples where the method has results with a very low positional accuracy. As can been seen in the figures, the sidewalk geometries overlap with the nearby buildings. This is a result of: (a) the lack of accuracy of GPS points in that street (due to the multipath error of GPS devices); and (b) lack of enough GPS points to select for construction of the network. Please note that our method aims to select at least one significant point every 5 m, and hence, for areas where fewer GPS points are available, even though the point does not fall into the acceptable range between road center and building footprints, it would still be selected for sidewalk construction. It is important to note that Google Maps does not necessarily represent the ground truth. The positional accuracy of imageries in Google Maps can vary a lot in different parts of the world and the image can be out-of-date. Therefore, for a better assessment of positional accuracy of the generated sidewalks, we performed a comparison with ground truth data (Section 4.3.2).

#### 4.3.2. Comparison with Sidewalk Reference Data

For this study, two spatial data quality elements were selected to be assessed: positional accuracy and completeness. Positional accuracy is the best established indicator of accuracy in mapping science [60] and therefore must be tested. When evaluating the fitness-for-use of data generated by GPS traces, the importance of positional accuracy is significant since the raw data were not created by professionals and without stringent data-collection standards. Secondly, Haklay emphasizes that completeness is significant in the case of VGI when data collection is carried out by volunteers [6]. To better estimate and analyze the positional accuracy as well as the completeness of the generated sidewalk geometries an extrinsic quality analysis of sidewalk network constructed by our method with comparison to a reference dataset of sidewalks from the municipality of Heidelberg is performed. Table 1 shows the results of the extrinsic quality analysis with two different indicators: the total length ratio of sidewalk geometries (division of total length of generated sidewalks to reference data) was computed to indicate the completeness level, and root-mean-square error (RMSE) value at the 95% confidence level was calculated to assess the positional accuracy of the results. The reason that the buffering approach suggested by Haklay [6] was not used is because implementing their method is more complex and less efficient to be performed for the small area of our experiment, although for bigger cities it is one of the best methods to evaluate the positional accuracy of features. To calculate the RMSE, a head-to-head positional accuracy assessment between generated sidewalk network and the sidewalk reference data was conducted using the approach presented in [61]. RMSE value is determined from the distances between the starting and ending points of the derived sidewalk vector data and their corresponding nodes in the reference data vector (ground truth) (i.e., absolute positional accuracy [62]). This type of comparison provides a quantitative way to describe the positional accuracy of the derived dataset. However, to prepare the dataset for comparison, it is required to perform a map matching process where two geo-referenced datasets (the generated sidewalks and the reference dataset) are geometrically overlaid. We followed the same method described in our earlier work [12] which in turn is adopted from the map matching algorithm proposed by [59].

**Figure 11:**
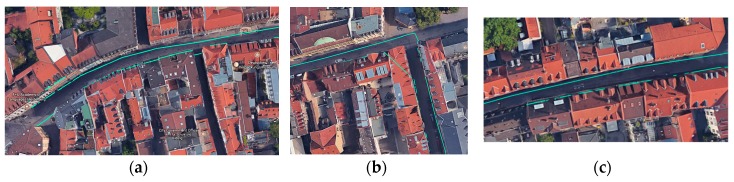
Results of visual inspection of sidewalk network with Google Maps (after applying the enhancement stage): (**a**–**c**) the same areas in Figure 10b–d, respectively.

In terms of evaluating the enhanced sidewalk network, we performed the same evaluation procedure. The results of visual analysis with Google Maps for sample areas with high positional error are depicted in Figure 11. Please note that these areas reflect the same areas depicted in Figure 10b–d.

The results of completeness assessment show that at the macrolevel, the total length of generated sidewalk network is 3974 m, while for the same network in the ground truth dataset it is 4225 m. thus, the generated dataset total length is 0.96 of the reference. The results of positional accuracy assessment show a high RMSE value calculated for the derived sidewalk network which shows that the original sidewalk is relatively inaccurate, while the enhanced version of sidewalk geometries is much more accurate. Furthermore, Table 1 shows the results of the extrinsic quality analysis of enhanced sidewalk network compared to the prior sidewalk network.

## 5. Discussion and Conclusions

Availability of sidewalk network data is mandatory in a variety of applications, especially in wheelchair routing and navigation services as well as urban planning projects. According to our investigation, sidewalk geometries are not available for all cities/countries in OpenStreetMap (especially for the four pilot sites of CAP4Access project: London, Heidelberg, Vienna, and Elche). However, some features have been mapped in some particular cities/areas, and, according to several forum discussions, the final interest and decision of the OSM community is to tag sidewalk information to road features rather than having the sidewalk geometries mapped directly. Hence, since sidewalk geometries are not available in OpenStreetMap database for our pilot cities, to prepare OSM data to be used for a wheelchair routing service, it is required to construct sidewalk geometries from available sources. This paper presented an approach to extract the geometry of sidewalk path segments and to construct sidewalk networks using multiple GPS traces. The algorithm is composed of four main steps (followed by an enrichment step): pre-processing and cleaning; data clustering and significant point filtering; map matching and candidate point selection; and sidewalk network construction. The algorithm was tested using GPS datasets collected by wheelchair users in the field. Based on the evaluation results, the RMSE value of the initial constructed sidewalk geometries is 3.2 m. This is a rather low positional accuracy especially for sidewalk routing services in densely populated areas. To improve the results, we presented a simple additional task of shifting selected significant candidate points to an acceptable range of sidewalks between roads and building footprints. This task can be performed only in areas where complete information about road and buildings are available in OSM. The RMSE value of the enhanced sidewalk network greatly decreased to less than a meter, resulting in a much higher positional accuracy. Furthermore, we calculated an index for evaluating the data completeness level of the results. This index was the ratio of total length of generated sidewalk compared to total length of sidewalks in the reference dataset.

The results show that for both original and enhanced results our approach seems to be acceptable and reliable for use in routing services where length of paths are important. Note that the length of the path is only one important factor for efficient wheelchair navigation. Further considerations are: (1) presence of a curb cut (roadway access point); (2) presence or enrichment of crosswalks [63]; (3) the running slope and (more critically) the cross slope of the walkway; and (4) walkway surface materials. Hence, future research study needs to be done for developing methods to collect and enrich attributes of sidewalks such as sidewalk width, incline, surface texture, etc. 

Based on the evaluation results, it is concluded that the algorithm can automatically construct sidewalk networks using multiple GPS traces. It is highly predicted that the number of GPS traces and the positional accuracy of the generated pedestrian path segments are positively correlated. Hence, more GPS data could potentially lead to more accurately positioned sidewalks. In principle, a low quality in terms of positional accuracy of sidewalks might also be introduced by a low positional accuracy of the OSM buildings and road datasets. Even though it was not the purpose of this paper, a preliminary check of this accuracy is suggested to be performed. Moreover, the assumption that GPS traces represent the sidewalk travelled is not always true. Deviations from a sidewalk are very common in an urban setting, and are caused by wheelchair users needing to travel in the roadway around obstacles. These deviations (and their causes) are important to explore. Finally, this study can be extended by checking the possibility of applying the workflow for smartphone location data. Employing smart phone location data or any other GPS traces (such as the ones uploaded by volunteers in OSM) to be used for sidewalk network construction is a potential research topic, given the fact that one needs to provide a solution on filtering points that do not belong to a sidewalk (e.g., when a user crosses the road in the middle of a street).

## Figures and Tables

**Table 1:** Result of extrinsic quality analysis.

Initial Experiment Results		Enhanced Results	
Total Length Ratio	RMSE (m)	Total Length Ratio	RMSE (m)
0.94	3.2	0.96	0.93
